# Distinguishing Syntactic Markers From Morphological Markers. A Cross-Linguistic Comparison

**DOI:** 10.3389/fpsyg.2020.02082

**Published:** 2020-08-18

**Authors:** Constanze Weth

**Affiliations:** Department of Humanities, University of Luxembourg, Luxembourg, Luxembourg

**Keywords:** syntactic marker, syntactic spelling, syntactic reading, English, French, Dutch, German

## Abstract

This brief review summarizes findings about syntactic markers, i.e., graphemic elements that indicate syntactic relations, such as inflection morphemes. Current spelling models subsume inflection with derivation and stem alternations under “morphological spellings.” They hence consider inflection only in relation to the orthographic word. This paper argues that syntactic markers are a specific category as they are part of the orthographic word but also systematically tied to the presence of syntactic features above the word level. Syntactic spelling refers thus not only to the correct spelling of a syntactic marker but to its correct application within a given syntactical context. In syntactic reading, (proof)readers must notice the marker and interpret it correctly to understand the sentence. Syntactic spelling and reading have hence been found to be highly demanding in many languages. Syntactic information is not decisive for sentence understanding in many cases, since the information can be deduced from the context. In order to focus the definition of syntactic markers, this paper restricts them to those graphemic elements that convey syntactical but no lexical features and are further unrelated to phonology. The paper concludes that syntactic markers and spelling should be distinguished from morphological spelling. Examples are given for English, French, Dutch, and German.

## Definition of “Syntactic Markers”

Syntactic markers are serial graphemic elements that indicate syntactic features. These features create coherence within phrases and between words or word groups on the clause level. Syntactic features are, therefore, not word-related but link larger entities of a sentence. In many languages, syntactic features are identical with inflection affixes. An example of this is conjugation: In English, the 3rd person singular is marked syntactically, distinguishing (I/you/we…) *sing* and (s/he) *sings*. In French, conjugation more strongly differentiates between the markers of person. However, only the 1st and 2nd person plural are phonologically transparent. All other persons differ in spelling but not phonologically (cf. for the verb *to sing* the 1st and 2nd person plural compared to all other grammatical persons: [ʃɑ̃te], [ʃɑ̃tɔ̃], [ʃɑ̃t]).

Another example is the nominal plural <s> in English: Pronounced [s], as in *cats* [kεts], the marker is phonologically transparent. Confusion might arise, however, between the ending of the (one-morpheme) word *fox* [fɔks] and the (two-morpheme) word *socks* [sɔks]. Moreover, plural <s> can be articulated as [s] or [z], depending on the previously articulated phonemes (Kemp and Bryant, 2003).

Neither all syntactic features, nor all markers, indicate inflection. Some mark a particular word class. The <wh> spelling in *whether*, for instance, highlights the interrogative pronoun in the paradigm of *what, when*, etc., and is therefore a syntactic marker. The homophone *weather*, in contrast, does not include any syntactic features. Similarly, in German, nouns and syntactic nouns are all spelled with an initial capital letter that highlights this word class in contrast to verbs and adjectives.

While many syntactic markers consist of a grapheme and represent a morpheme, such as plural <s> in English, they might consist of a grapheme that is not related to a separable morpheme, such as <wh> in interrogative pronouns. In some cases, it is even difficult to define the grapheme status of a syntactic marker, such as in the capital spelling of nouns in German (Kohrt, 1985). A difficult graphematic status is also found with the apostrophe, distinguishing between the possessive < ′ > or <’s> (case) and the plural <s> (numerous), as in *cat’s – cats’ – cats* [kεts] (Bunčić, 2004).

Punctuation is not included in the definition of syntactic markers and hence not part of this paper. Simply put, punctuation refers to the global sentence structure, whereas syntactic markers refer to local contexts below sentence level, such as noun phrases.

Syntactic spelling refers not only to the correct spelling of a syntactic marker but to its correct application within a given syntactical context. This has been observed as highly demanding in several languages such as English (Kemp et al., 2017), French (Fayol et al., 2006), Dutch (Sandra et al., 1999; Bosman, 2005; Sandra and Van Abbenyen, 2009), German (Betzel, 2015), and Greek (Protopapas et al., 2013). Only phonologically inaccessible syntactic markers seem to be particularly difficult to spell.

This paper proposes, therefore, to define “syntactic markers” as graphematic elements whose occurrence is systematically tied to the presence of syntactic features. As the spelling of syntactic markers is particularly demanding when these markers are not phonologically deducible, the following considerations focus on these syntactic markers. Examples will be provided across English, French, Dutch, and German.

## Syntactic Markers Across Orthographies

In English and French, as well as many other languages, syntactic markers are inflection suffixes that indicate agreement or government on the level of phrase or clause. However, syntactic features differ between languages and in some cases, such as German, syntactic markers refer neither to inflection, nor to any other specific morpheme. The following examples of syntactic markers indicate syntactic relations and share the common feature that they cannot be inferred from the phonological structure.

A syntactic marker famously prone to spelling errors in **English** is the past tense marker on regular verbs <ed> such as *kissed* (Nunes et al., 1997b). The marker clearly indicates a verb form in contrast to nouns or adjectives. The phonological word form varies, according to the phonological context, between [t] or [d]. Confusion in spelling might be possible between the ending of a (one-morpheme) noun such as *bird* [bǝrd] or *belt* [bεlt] and the (two-morpheme) verb *called* [kɒld] or *dressed* [drεsd]. While the past tense of each regular verb is spelled <ed>, irregular verb forms are phonologically more transparent by deleting the silent <e>, such as *found* [faʊnd] and *felt* [fεlt].

In oral **French**, the singular and plural sound identical, except of the article: *Le grand chat noir mange* [lǝ gʁɑ̃ ʃa mɑ̃ʒ] vs. *Les grands chats noirs mangent* [le gʁɑ̃ ʃa mɑ̃ʒ] (“The big cat/s black eat/s”). The plural marker has two forms: <s> for adjectives and nouns, and <nt> for verbs (3rd person plural). The singular form is not marked orthographically. Importantly, plural is conveyed by all the elements within a noun phrase and within subject-verb agreement (Dubois, 1965). Other syntactic markers that are extremely difficult to distinguish in spelling are the forms <er, ez, é, ée, és, ées, ai, ait, ais>. Each marker conveys precise information about person and/or number of nouns and adjectives, or various conjugations of verbs and participles. All markers are pronounced equally as [e] (Brissaud and Chevrot, 2011).

While homophony is the default in French inflection, it concerns only a small part of verbal inflection in **Dutch**. Present and past tense have a regular inflection pattern with stem + suffix. In present tense, the 1st person singular keeps the stem form, the 2nd and 3rd person singular add the suffix <t>. In most cases, both verb forms are phonologically transparent. They become homophonous, when the stem ends on <d>, i.e., *vinden* (“to find”), *vind* (1S), *vindt* (3S), both pronounced as [vɪnt]. In past tense, suffixes are for singular <de> (or <te>), for plural <den> (or <ten>). While in most cases the spellings are phonologically transparent and distinguish the stem in the first and the suffix in the second syllable (*belde* ([bεl.dǝ], “called”), stems ending on <d> (or <t>) mask this syllable structure as both <d> (or <t>), from the lexical stem and the suffix, are represented (cf. *meldde* [mεl.dǝ], “to inform”). Homophone dominance, on the lexical and sublexical level, increase congruity errors on the lower-frequency form (Sandra and Van Abbenyen, 2009).

Whereas in English, French and Dutch, inflection suffixes are syntactic markers, **German** syntactic markers do not necessarily point to inflection, nor do they always refer to a morpheme. One syntactic marker signifies the word class “noun” or, more precisely, the head of a noun phrase (NP) by an initial capital letter. Indeed, almost every word can become a noun without any morphological modification, although this is mainly applied to adjectives and verbs. An example for a verb vs. a nominalized verb is *Ich hörte sein Singen* (“I heard his singing”) vs. *Ich hörte ihn singen* (“I heard him singing”).

While the lexical-semantic characteristics of a noun are not clear-cut but lie on a continuum between a prototype and its periphery, the syntactic context of the noun phrase remains stable: In this perspective, capital spelling applies to the head of a NP. Whether a word is head of the NP is shown by whether the adjectives, with which the NP can be extended, are inflected. An adjective such as *schön* (“nice”) can be used in this uninflected form at several positions in the sentence, e.g., *Ich hörte ihn schön singen* (“I heard him singing nicely”). However, it must be inflected within the noun phrase, as in *Ich hörte sein schönes Singen* (“I heard his nice singing”) (Funke, 2020). While the noun closes the NP-unit, the capital letter highlights this demarcation visually (Maas, 1992).

These non-exhaustive examples in French, Dutch and German illustrate the definition of syntactic markers. The general scheme of French agreement reveals the relational aspect of these markers, as they have to be placed, redundantly, on each word of the syntactic unit (phrase or clause). The low occurrence of the Dutch examples reveals “homophone dominance” effects (Sandra and Van Abbenyen, 2009, p. 243; cf. Largy et al., 1996). The German examples show that a syntactic marker might not be classifiable as morpheme or grapheme (Kohrt, 1985), nevertheless, the capitalization of the noun is the visual index of a syntactic unit.

## Syntactic Spelling

All existing spelling models have focused on the orthographic word. This is consistent, as all orthographic regularities are word-based. Early spelling models described spelling acquisition as a linear process in which learners first discover relations between graphemes and phonemes, and subsequently acquire orthographic and morphological structures represented in the respective writing system (cf. Frith, 1985). More recent approaches to spelling such as the triple word-form theory (Garcia et al., 2010; Bahr et al., 2012), have shown that learners do not acquire the linguistic levels coded within a writing system linearly. Instead, spelling development is a long-term process during which learners must learn to coordinate the different layers of the writing system (Sprenger-Charolles et al., 2003; Bahr et al., 2012). Existing spelling models distinguish between phonologic, orthographic, and morphological spellings.

So-called “morphological spellings” (Pacton and Deacon, 2008; Bahr et al., 2012) refer to morphologically complex words with stem (e.g., *sing*) and one or more affixes, and enclose derivational (e.g., *singer*) and inflectional (e.g., *sings*) morphology. It is suggested that inflection might be easier as derivation as young children typically focus on inflection (Carlisle, 1996; Kirby et al., 2011) and as the rules for inflection suffixes are, in general, very easy (such as 3rd person singular <s> in English). Therefore, authors rather point to inflection errors of young learners when those show over-generalizations of regular spelling such as *snowmans instead of *snowmen*.

Surprisingly, syntactic spelling refers to the regular forms and is based on a rather simple abstract, general rule. Although young spellers already identify, and may correctly produce, syntactic markers (Totereau et al., 1997; Turnbull et al., 2011), many studies have shown that learners’ difficulties with syntactic markers may persist throughout school (Bryant et al., 2000; Totereau et al., 2013; Betzel, 2015). Even literate adults may produce syntactic spelling errors, observed in experiments (Largy et al., 1996) and in naturalistic writing situations (Surkyn et al., 2019). Indeed, the correct detection or production of a syntactic marker is not a result of the lexical identification of a word, but of structural relations within a group of words (Bock and Ferreira, 2014). The relational characteristics of syntactic markers – and the difficulties in processing them – become apparent in studies that analyze syntactic processing in spelling and reading.

Known sources of syntactic, or, more precisely, congruity errors are the effect of frequency and analogy, especially on the spelling of homophonous word forms, and the effect of words in the proximity of the target word. Resulting from experiments in French and Dutch, working-memory seems to be an important triggering factor for the emergence of congruity errors in homophones (Fayol et al., 1994; Largy et al., 1996; Sandra and Van Abbenyen, 2009). Sandra and Van Abbenyen (2009) additionally, suggests the importance of the process of lexical access in the long-term memory, assuming that storage of a given inflected verb form as well as the occurrence frequency. This is in line with the observation that younger learners seem to store some inflected words in the orthographic lexicon, as they experience them more frequently than others (Largy et al., 2007; Geoffre and Brissaud, 2012).

More specifically, the experiments in French have shown that subject-verb agreement errors occur when the agreement between subject and verb is covert. Prototypical examples are sentences with a subject containing two noun phrases mismatched in number (_clause_[_NP–Sg_[The girl] _PP_[of _NP–Pl_[the neighbors]] sings]). While the first NP is the subject-NP, the second NP is a modifier of the subject-NP. If a second task needs attention, even literate adults do not always refer to the syntactic relation while spelling but tend to automatically produce syntactic markers between the NP adjacent to the verb (_clause_[_NP–Sg_[The girl] _PP_[of _NP–Pl_[the neighbors]] *sing]). Fayol and colleagues have interpreted these attraction-errors (Bock and Miller, 1991) as a by-product of the automatization in syntactic spelling (Fayol et al., 1994). For learners, maintaining in memory the sentence to be written might be enough to disrupt the control for agreement (Fayol et al., 1999).

Other experiments were concerned with congruity errors in spelling verbal inflection with homophone nouns and verbs with different frequencies. Homophones were elicited in syntactic ambiguous (Largy et al., 1996) and unambigous contexts (Sandra and Van Abbenyen, 2009). In both experiments, congruity errors increase in adults and young learners when the noun is more frequent compared to the verb. The same effect was shown on sublexical level with concurrent word final spelling (Sandra and Van Abbenyen, 2009). The homophone dominance effect occurs on time pressure or under the condition of a secondary task. Observations on the development of the numerous alternative forms of the homophonic word ending [e] confirm the causally involved long-term memory and working memory. Development entails first the acquisition of the markers itself and its overgeneralization, then an increase of correct agreement, and from mid-secondary school on a decrease in agreement errors (Brissaud and Chevrot, 2011). The authors attest further that experienced writers also may recur to the most frequent word form under time pressure or in demanding writing contexts.

### Syntactic Reading

Experiments on the detection of linguistic in/congruency while reading strengthen the results on spelling. On the basis of reaction time in a negative priming study, the observed effects reveal the executive costs of activating the strategy that a French NP requires <s> inflection after a plural determiner (Lanoë et al., 2016). The authors suggest the relevance to inhibit a highly automatized but in a given context misleading strategy that is added to the needed activation of the correct inflection marker. Note that in a sentence such as *Je mange les bonbons* vs. Je mange les *bonbon (“I eat the sweets”), all tested age groups, 6 graders, 9 graders, and adults required more time to determine the correct plural inflection of a noun when the sentence was preceded by a sentence with the pronoun *les* (3rd person plural), homophonous to the plural article *les* (i.e., *Je les mange* “I eat them”).

Studies on adolescents’ proofreading of Dutch verb homophones, similarly, evoke inhibition of an overlearned spelling pattern (Verhaert, 2016; Verhaert et al., 2016). They observed that error rates on homophone congruency amounted with the frequency of the verb, suggesting, as for spelling, an effect of homophone dominance. Due to the similar results of homophone dominance in spelling and proofreading and referring to the persistence of errors in syntactic spelling, the authors indicate a double trap for spellers, first during spelling, then during re-reading (Verhaert et al., 2016).

The here presented studies focus on the detection of orthographic markers in a given syntactic context while reading. However, most syntactic features that readers encounter in texts are embedded in semantics and context. In the incorrect example *the friends house, the missing apostrophe does not hinder comprehension, as the construction can only be understood as a possessive. This would be different if the word after *friends* could be a nominal or verbal form, as in *the friends drink* vs. *the friend’s drink*. In first-pass reading, a reader will parse the syntactic structure embedded in the semantic context without necessarily identifying it. Syntactic reading takes place in cases of doubt or whenever the information cannot be extracted from the semantic context. In these cases, readers use the probabilistic cues to grammatical category at the beginning and end of a word (Arciuli and Monaghan, 2009). On this basis, readers take a lexical decision in sentence production and judgment (Kemp et al., 2009). An example of a syntactic reading task are parallel-constructed sentences where a syntactic marker is decisive for understanding. The study of Funke and Sieger (2012) asked pupils with perfect mastering of capital spelling of nouns to read sentences and then choose the correct ending of the sentence depending on whether a key word was a noun (i.e., capitalized) or a verb. A contextualizing sentence preceded each sentence. An example of the task is (Funke and Sieger, 2012, p. 1774):

Derek says, “Nowadays, so many people are divorced after only a few years of marriage. Most love

…… someone else after a while.”

…… ends sadly.’

The critical word in this example is *love*, used as a verb (solution a) or as a noun (solution b). In German, this difference is displayed in orthography as the noun would be capitalized. Although the participants were highly skilled spellers, only 30.7% of them reached the criterion of at least 15 (of 20) correct solutions in this task. More specific analyses revealed that pupils nevertheless seem to have considered capitalization while reading.

The presented research on syntactic spelling as well as syntactic reading indicates that syntactic spelling and proofreading might be similar processes (Verhaert et al., 2016). Both become conscious, hence non-automatic and slow when spellers or (proof)readers inhibit competing word forms associated with the linguistic context (Bock and Levelt, 1994). These processes differ greatly from the supposed automatic and fast visual word recognition process.

### Training of Syntactic Spelling in Typical Educational Environment

Syntactic markers belong to the domain of orthography, as they are word-bound, but indicate relational information on phrase and clause level. Training of these markers seems complex as the processing of syntactic markers does not seem to be a precondition for the accomplishment of first-pass reading and writing tasks. However, on the one hand, performant readers do use syntactic markers for reading (Kemp et al., 2009; Funke and Sieger, 2012). On the other hand, syntactic spelling is difficult for all writers and proofreaders.

Regarding teaching, some studies indicate that children seem to discover syntactic constraints on spellings, at least to some extent, without being explicitly taught (Nunes et al., 1997a; Funke et al., 2013). However, the input material and the studying task is crucial for a potential discovery of the syntactic structure by the learner (Funke et al., 2013). Few intervention studies have trained a narrowly defined syntactic marker. The following intervention studies have drawn explicit attention to syntactic markers and have reported training effects on spelling.

Training effects are reported for English past-tense spelling (Nunes et al., 1997a) and the apostrophe (Bryant et al., 1997), for French plural markers (Thévenin et al., 1999; Bîlici et al., 2018) and for German (Bîlici et al., 2020). A training that focused on noun phrases in a sentence found effects on capital spelling of nouns, even if controlled with a group that focused on the lexical category noun (Brucher et al., 2020).

## Discussion: Modeling the Processing of Syntactic Markers

This review provided new perspectives on a category of orthographic markers that relate to syntax. Syntactic markers are the interface between orthography and syntax. Clearly, the syntactic marker is part of the orthographic word and might be stored, as part of the inflected word form or as suffix, in the orthographic lexicon. However, it refers to structural information on phrase and clause level.

Interestingly, all presented syntactic markers are based on very simple rules such as “if nominal plural add <s>” or “if noun use capital letter.” These rules are part of the curriculum since the beginning of primary school. The learning process of syntactic markers seems confusing at first sight: While young spellers already identify and may correctly produce syntactic markers, even highly literate adults commit spelling errors in certain spelling tasks. This may be due to the fact that syntactic markers are, in most cases, redundant with phonology, semantics or context. In these cases, it is irrelevant whether a reader or writer notices and correctly interprets or produces the syntactic form-function relationship. In ambiguous syntactical contexts, however, syntactic spelling and reading is highly demanding and leads to rare but systematic errors, even in adults.

Several of the here quoted authors have proposed a model, that describes the processing of syntactic markers. The authors agree that learning of syntactic markers relies on the acquisition of the declarative spelling rules and activation of the correct inflection. They also agree that errors in experienced writers may be a by-product of the automatization of these rules.

Sandra and Van Abbenyen (2009) assume a full-form representation of inflected word forms in Dutch as well as two memory systems that might be causally involved in errors of syntactic markers: a given verb form and its occurrence frequency in the long-term memory as well as the conscious rule application of verb homophones in the working memory. Limitations of the working memory under conditions of time pressure or a secondary task lead to the homophone dominance effect. While in Dutch the application of syntactic rules for verb inflection applies only in a minority of cases, it seems also warranted for French where homophone inflection is the rule, not the exception (Largy et al., 1996). On the basis of priming studies on the detection of French plural markers, Lanoë and colleagues (2016) emphasize the ability to inhibit the overlearned strategy in order to select the syntactic marker associated with the linguistic context among homophone concurrent forms. All descriptions emphasize that the particular difficulty lies in choosing the right word-form amongst several competing word forms. This is even more difficult if the syntactic context is covert, such as NP1+NP2+V-sentences (Bock and Miller, 1991).

On the basis of the reviewed research, this paper emphasizes that syntactic markers and processing should be clearly distinguished from morphological spelling. Furthermore, it proposes limiting the category of “syntactic markers” to elements that convey structural-relational but no lexical features and that are either unrelated to phonology or cannot be recoded clearly. This heuristic limitation serves to distinguish the difficulties in processing syntactic markers systematically, as they are both syntactic and not supported phonologically. This is crucial to improve our understanding of the causes of spelling difficulties related to syntactic markers as well as the relation between orthographic form and syntactic function across languages.

## Author Contributions

CW was the sole author and was responsible for all sections of the manuscript.

## Conflict of Interest

The author declares that the research was conducted in the absence of any commercial or financial relationships that could be construed as a potential conflict of interest.
